# Myofibroma of the nasal vestibule: a case report and review of the literature

**DOI:** 10.1093/jscr/rjaf969

**Published:** 2026-01-15

**Authors:** James E Holland, Emily Lowe, Karwan Moutasim, Badri Srinivasan, Rami J Salib, Huw A S Jones

**Affiliations:** Department of Otolaryngology/Head & Neck Surgery, University Hospital Southampton, Tremona Road, Southampton, Hampshire, SO16 6YD, United Kingdom; Department of Otolaryngology/Head & Neck Surgery, University Hospital Southampton, Tremona Road, Southampton, Hampshire, SO16 6YD, United Kingdom; Department of Histopathology, University Hospital Southampton, Tremona Road, Southampton, Hampshire, SO16 6YD, United Kingdom; Department of Oral & Maxillofacial Surgery, University Hospital Southampton, Tremona Road, Southampton, Hampshire, SO16 6YD, United Kingdom; Department of Otolaryngology/Head & Neck Surgery, University Hospital Southampton, Tremona Road, Southampton, Hampshire, SO16 6YD, United Kingdom; University of Southampton, University Road, Upper Shirley, Southampton, Hampshire, SO17 1BJ, United Kingdom; Department of Otolaryngology/Head & Neck Surgery, University Hospital Southampton, Tremona Road, Southampton, Hampshire, SO16 6YD, United Kingdom

**Keywords:** myofibroma, myofibromatosis, nasal vestibule, nasal ulceration

## Abstract

Myofibroma is a rare benign myofibroblastic tumour of spindle cell differentiation. Most commonly, myofibromas are observed in the context of a myofibromatosis disorder in children. Rarely, myofibroma can present as a solitary lesion in adults. We report a rare case of solitary myofibroma of the nasal vestibule which presented as a nonhealing ulcer on the anterior nasal septum. This is described in the context of a comprehensive literature review. Punch biopsies were obtained which revealed histological appearances in keeping with myofibroma. Following discussion at the regional head and neck and sarcoma multidisciplinary meetings, surgical excision was performed with staged reconstruction. To our knowledge, this is the first reported case of myofibroma arising from the anterior nasal septum and columella. This case highlights the importance of considering myofibroma as a differential diagnosis for nonhealing ulceration in this anatomical location.

## Introduction

Myofibromas are rare benign tumours of mesenchymal cells exhibiting myofibroblastic differentiation [1]. Myofibromas typically appear as slow-growing painless masses, most frequently in the skin, subcutaneous tissue, bones, and skeletal muscles of the head and neck (H&N) [2]. Diagnosis relies on tissue biopsy and thorough histopathological examination. This will typically characterize a biphasic pattern of growth with alternating areas of primitive cells showing necrosis/calcification and discrete nodules of spindle cells [3, 4].

An estimated 90% of myofibromas present before the age of two. These occur most frequently as part of a myofibromatosis disorder [5]. However, myofibromas can rarely manifest as a solitary lesion in adults. The WHO 2020 classification of soft tissue tumours recommends the use of terms myofibroma and myofibromatosis to differentiate between adult solitary and infantile multicentric subtypes, respectively [6]. Myofibromas are thought to arise sporadically; however, there is emerging evidence for autosomal dominant and recessive patterns of inheritance in infantile myofibromatosis [7–9].

Solitary H&N myofibromas are rare, but most frequently arise as a nodule on the skin of the H&N and oral cavity (buccal mucosa/gingiva) [10]. This case describes a solitary adult myofibroma presenting as an ulcer in a highly unusual anatomical location within the nasal vestibule. Although benign, myofibromas have the potential to cause local tissue destruction and deformity, so are typically managed with surgical resection.

## Case report

**Figure 1:**
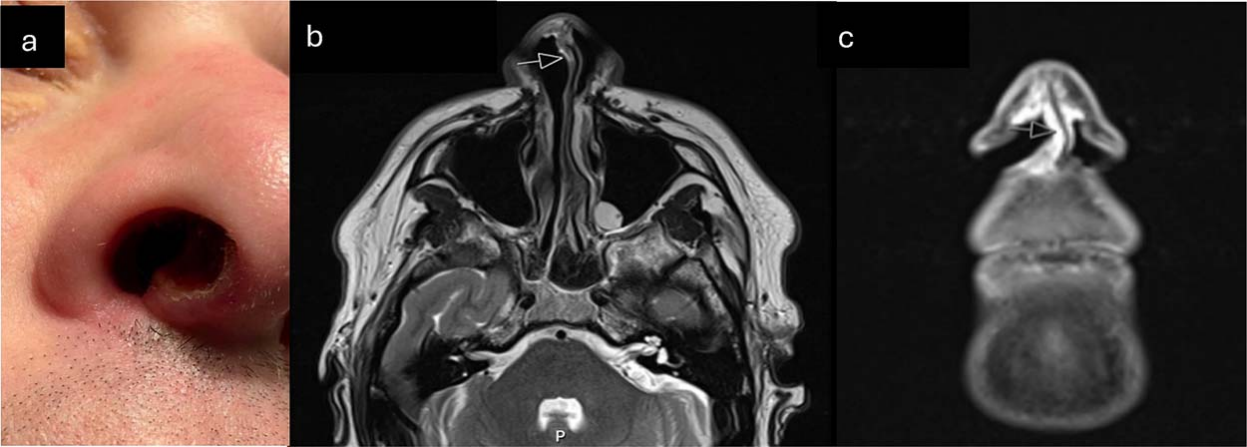
(a) Clinical photograph demonstrating ulcerated lesion of anterior membranous nasal septum (b) T2 axial MRI-arrow indicates soft tissue thickening of columella (c) Coronal STIR MRI sequence—high signal soft tissue thickening extending from columella to right nasal sill (indicated by arrow).

A male in his 30s was referred to our tertiary Rhinology unit with a 6-month history of a nonhealing right nasal vestibule ulceration (Fig. 1a). He described right nasal obstruction, pain radiating to the right eye, occasional serosanguinous rhinorrhea, and intermittent cacosmia. He denied epistaxis or taste disturbance. He was referred from the community after an unsuccessful trial of conservative management with oral and topical antibiotic. He denied weight loss and was otherwise well, with no rhinological history of note. He denied alcohol consumption and previously smoked cigarettes.

Anterior rhinoscopy demonstrated a 1 cm ulcer on the right anterior membranous septum, extending posteriorly to the cartilaginous septum and superiorly under the upper lateral cartilage. The edge was rolled, crusty, and had a sloughy base overlying visible perichondrium. Rigid endoscopy revealed no further local progression, and no other lesions of concern. There was no cervical lymphadenopathy.

**Figure 2:**
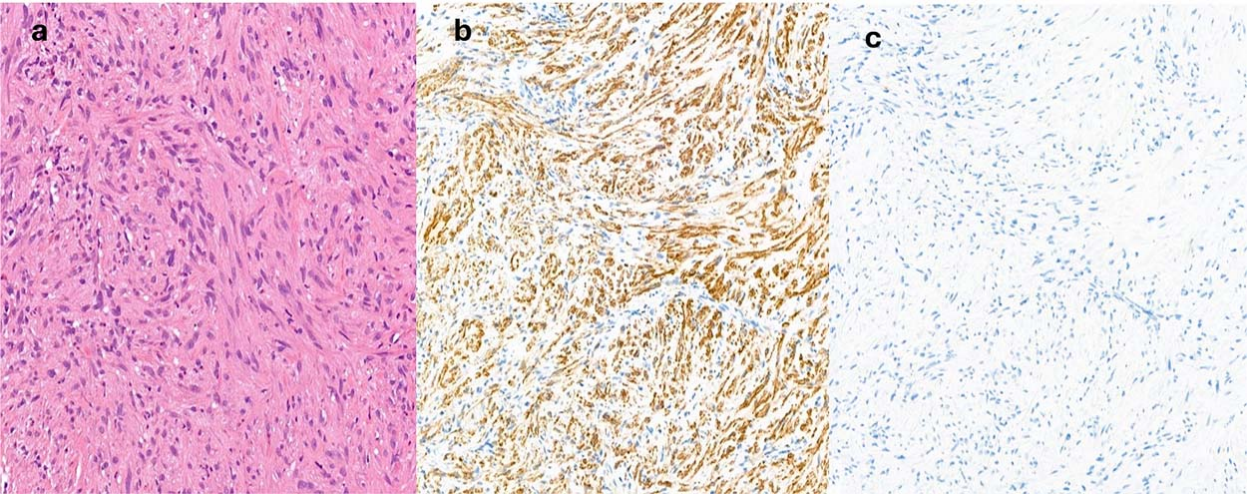
(a) Haematoxylin and eosin (H&E) sections show a spindle cell proliferation with no discernible atypia, short fascicles of cells with spindled nuclei with some vague zonation, SMA immunohistochemistry is positive (b), whilst desmin (c) is negative.

**Figure 3:**
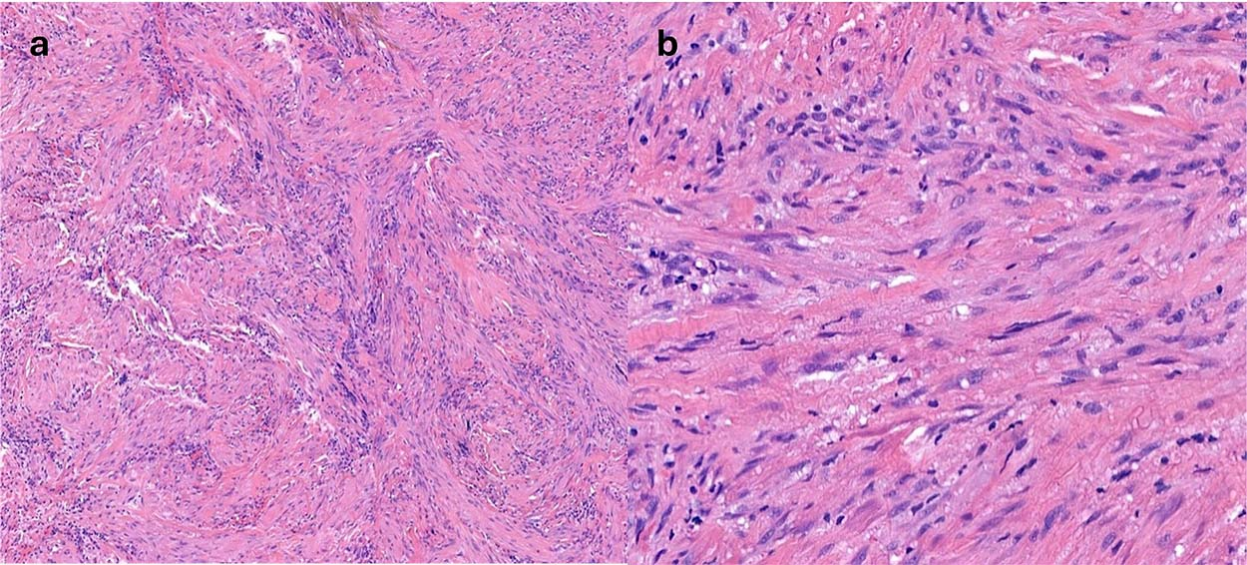
(a) H&E stain of surgically resected specimen (b) high powered image of surgically resected specimen showing nuclear detail.

Punch biopsy was obtained under local anaesthetic. Computed tomography was obtained initially, followed by Magnetic Resonance imaging (MRI) (Fig. 1b and c). These demonstrated an abnormally enhancing soft tissue thickening involving the right alar rim, columella, and undersurface of the anterior nasal vestibule. Histological appearances were suggestive of myofibroma but given the clinical and radiological context, differential diagnosis also included low-grade myofibroblastic sarcoma (Figs 2 and 3).

This case was discussed at both H&N, and Sarcoma multidisciplinary team meetings, and recommendation was for surgical excision with staged reconstruction. Excision of the lesion was performed, and subsequent re-excision was required on two occasions due to positive margins. All subsequent histological assessments were in keeping with myofibroma.

## Discussion

Ulceration of the nasal septum presents a significant diagnostic challenge for ENT specialists. Differential diagnoses include infections (e.g. tuberculosis, leishmaniasis, leprosy, and rhinoscleroma), inflammatory conditions (vasculitis, Churg-Strauss syndrome, and lupus erythematosus), and malignancy (squamous cell carcinoma and NK/T-cell lymphoma) [11, 12]. Accurate and timely diagnosis relies upon thorough clinical assessment and early histopathological examination. Prolonged periods of observation can delay the diagnosis of neoplastic disease.

Myofibromas are pericytic/perivascular soft tissue tumours with overlapping histological features with myopericytomas, glomus tumours, and angioleiomyomas [3, 13, 14]. Given similar histological features, these are often considered part of the same disease spectrum [15, 16]. Another important differential for a low-grade myofibroblastic proliferation in the H&N region is low-grade myofibroblastic sarcoma [17]. Given overlapping features, careful histological examination is essential. There is a potentially a role for immunohistochemical (h-caldesmon, desmin, smooth muscle actin, and muscle-specific actin) and cytogenetic analysis (SRF-RELA, NOTCH, and PDGFRB genes), but these techniques lack absolute specificity [3, 13, 18–20]. RNA next-generation sequencing was attempted in this case but no fusions (e.g. SRF-RELA) were detected.

A comprehensive literature review was performed to identify relevant case reports. Walsh *et al*. [21] describe two cases of nasal myofibroma: (i) a solitary infantile nasal myofibroma arising from the inferior turbinate and soft tissues of the nose/cheek in an 11-year-old male and (ii) a male in his 50s with a myofibroma originating from the lateral wall of the nasal vestibule. Notably, both presented as solitary nodules and stained positive for smooth muscle actin and negative for desmin. Both were excised, with no evidence of recurrence at three years and one year, respectively. Josephson *et al*. [22] described an infantile solitary myofibroma of the anterior skull base in a 7-month-old child, and Swaika *et al*. [23] reported a solitary infantile myofibroma of the posterior ethmoid sinus/anterior skull base in a 1-year-old with intracranial extension. Similar tumours with overlapping histopathological features, such as glomus tumours, glomangiomas, dermatomyofibroma, or haemangiopericytoma have also been reported in the nasal septum and vestibule [24–28].

## Conclusion

This case represents, to our knowledge, the first report of an adult solitary myofibroma of the nasal vestibule presenting as an ulcerative lesion. Key learning points include the need for early biopsy in persistent nasal ulceration to exclude neoplastic disease and the importance of recognizing myofibroma as a differential diagnosis for a benign spindle cell lesion in this location. Complete surgical excision is necessary given the potential for progressive local tissue destruction in a cosmetically sensitive area of the midface.
